# A Consideration of the Causes of Dental Disease with Special Reference to Present-Day Practice

**Published:** 1915-08

**Authors:** Otto E. Inglis

**Affiliations:** Philadelphia


### A CONSIDERATION OF THE CAUSES OF DENTAL DISEASE WITH SPECIAL REFERENCE TO PRESENT-DAY PRACTICE.

BY OTTO E. INGLIS, D.D.S., PHILADELPHIA.

Much intelligent thought is being applied by the deeper thinkers of our profession to the question of the relation of systemic conditions to the diseases of the teeth and associate parts. A careful search, however, shows that there is much confusion as to the actual relation between any given systemic condition or disease and given dental disease, for example, dental caries or pyorrhoea alveolaris. We may take for a further example the difficulty experienced in proving the relation of sulpho-cyanides in the saliva to dental caries, of syphilis, tuberculosis, gout or intestinal disturbance to pyorrhoea alveolaris. We have grown accustomed to associate susceptibility with dental caries and pyorrhoea, and it seems at times, and especially in aggravated cases, as though these must be. Yet in this paper, and at the risk of a charge of lack of breadth of view, I am impelled by a sense of justice to express the feeling that in going far afield in search of underlying causes we have developed a sense of hopelessness in our coping with cases of long-standing and aggravated nature. It seems to me that in such cases we are inclined to overlook conditions which should be obvious upon careful examination.

We can not enter a denial of the fact that many patients seem subject or susceptible to dental caries, and we have sought many explanations. Because we find in the patient some systemic defect are we justified in assuming that that defect is the cause of the caries, or if we treat systemically for that defect in conjunction with local treatment, are we justified in ignoring the benefits of the latter and in giving all credit.

As an illustration of the type of mind which performs this let me cite the case of a woman possessed of fine teeth almost excessively brushed frequently with consequent slight gum recession, the gum margin apparently healthy except for such slight retraction. Her teeth were completely attended to, and after an absence of a year or more she returned, saying that all her fillings were coming out, and that her teeth were sensitive upon the use of the brush. Before the patient took the chair I expressed my surprise, and said I hoped things were not as bad as she told me. A careful examination showed not a filling disturbed; not a single fresh cavity; very little calculus on the lower incisors, none elsewhere; no gingivitis, and merely one or two spots of cervical sensitivity entirely curable by the use of silver nitrate. Upon being so informed the patient exclaimed, "Doctor, do you know what has done this good work? It's the Celestine vichy I have been drinking every day since I left here." I ventured to suggest that perhaps the careful work I had previously done and the care she had given her teeth in the meantime might have something to do with the case, to which, with some reluctance, she agreed, but she probably still maintains her vichy attitude as a mental reservation.

No doubt some one will make the suggestion that Dr. Black, in his experiments with Epsom salts, apparently was able to limit the formation of the organic substance forming the basis of calculus, and which he called "agglutinin." Let us admit for a moment that the microbic plaque may possibly have a similar origin, the question arises why did this patient have some calculus. If so, why did not the vichy, taken constantly, stop the "agglutinin" formation; second, could not the alkalinity of the urine, and presumably by analogy the saliva, have introduced an element neutralizing any acid formed locally; third, did either of these possible effects have any more influence than the removal of local causes by friction. That brings up again our original proposition. Are we not overlooking the obvious?

About four years ago a lady came to me from the hands of an excellent operator. The mechanics of his filling was of the highest order. Yet after only a reasonable absence from his hands there was no difficulty in finding quite a large number of cavities. These were in due course filled, nothing at all obvious being left uncared for. The teeth were thoroughly cleansed and directions for care given which the patient followed to the best of her ability.

The patient being somewhat of a traveler, could be seen only about each six months, and at each visit since the first series of operations, very little work could be found and often only a cleansing necessary. When a cavity was found it invariably was at some spot difficult of access for cleansing by the patient. In addition, this patient has a rather frail constitution and teeth of poor quality. Many of the original cavities of decay were located in peculiar points of malformation even on the labial of incisors at the middle third of the crown, a position all will recognize as unusual.

It may be argued that the patient has lost a susceptibility previously possessed. If so, why does caries occasionally appear, and does susceptibility affect merely one or two teeth?

Suppose we grant a previous susceptibility was the condition in which I found her teeth, due to it or to the oversight of the previous operator, or her own unintelligent care of her teeth. I wish at this point to state that in my experience much of the ravages attributable to susceptibility are due largely to the fault of the dentist who does not obliterate all the discoverable cavities nor does he instruct the patient in the proper self-prophylaxis.

A speaker in referring to amalgam recently stated that if a defect were left at the cervix of a filling or subsequently occurred, the patient would have caries if a future period of susceptibility should set in. In my experience, if such a defect occurs at any point not covered by the gum, or not irrigated by the brush or floss, caries will almost always occur as a discoverable cavity in a reasonable period of time.

Apropos of this is my opinion that caries is a slow process, beginning with plaque formation, the germs in the plaque being nourished by carbohydrate food, lactic acid being a resultant product which causes decalcification, etc.

I believe that caries of the enamel up to the point of a discoverable cavity, is a question of a year or two at least in teeth of fair quality, perhaps less in those of poorer degrees of organization.

It is my view that this is the reason why prophylaxis performed each one, two or even three months by the dentist, or daily by the patient, or both in conjunction, is capable of preventing caries by constant removal of the plaques. Miller, in his book, describes the experiment of taking unusual bacteria of harmless character into the mouth. They disappeared very shortly. If now, the teeth are freed of microbic plaques, why should not lactic acid producing bacteria very shortly disappear, or if reintroduced, find great difficulty in finding a foothold.

I am accustomed to use an illustration of this point by describing a clump of toadstools on my lawn. One day I knocked them all over with my foot. A day or two later half as many new ones were growing in the same place. I repeated the process, and again about ten appeared. These were knocked over, and since that time, about four years ago, none have appeared. If now, we knock over the bacteria in the plaques, they require time to get into militant form again, so to speak, and then only if vigilance is relaxed. Just here in the defects of fillings, etc., above referred to, they accumulate and can not be removed even by persistent effort, hence their effects.

Dental caries then being a bacterio-chemical process, having several links in the chain, namely (1) inspissated mucin (possibly a special "agglutinin") which becomes the habitat of bacteria, thus forming a microbic plaque, (2) carbohydrate food, or for the sake of argument, we will admit, a glycogenic principle introduced via the saliva, (3) lactic acid as a product of bacterio-chemical action, (4) further action by bacteria acting on decalcifical tooth structure, or in other words, the organic matrix, and producing its putrefaction.

If we can eliminate any link in the chain we can cure the disease.

What evidence have we of the presence of these plaques or as I have here chosen to term them "the obvious?" First, we have the knowledge given us by Miller that bacteria of acid-forming character are always present in cavities of decay. Second, Williams has shown the plaques always present upon the surface of unbroken but carious enamel. Third, we can always find plaques of some sort at any unbrushed or unflossed portions of teeth upon even the teeth which to the eye are apparently clean.

Anyone can demonstrate this easily simply by painting a tincture of iodine over the teeth and immediately douching with water. The iodine is washed from the clean portions, but remains fixed in the plaques though the color soon fades away.

If floss is now passed between the teeth and worked against the plaques it will be seen that it is readily wiped off at all points except the middle of the cervix, labially and lingually.

Just here I would point out that this stain at the middle of the cervix just at the angle made between the tooth and the somewhat corded edge of the gum margin will not have been removed in many cases by patients who have brushed the teeth just before coming to the office. Those who have been vigorous and determined in the act of brushing may have cleansed some or even all teeth at this point, but almost invariably there will be found some gum recession as the result, and the further result that the gum margin becomes still more corded and the tooth still more difficult of subsequent cleansing. Abrasions from the vigorous use of powders are also frequently a result.

Let us now drive the point home. How many dentists have shown these plaques and demonstrated their removal to the patient?

It has been my experience to be almost invariably told by patients that they have never been shown before. I may state here that I have one patient who returned, asking for cleansing of the teeth only, frankly stating that my prices for filling were too high for her to pay, but that she had been unable to get any one she had consulted to cleanse her teeth in the manner she had had it done at my hands.

I hope I may not be considered as egotistically thinking that no one else does it, but simply as stating a condition that seems to exist, indicating a lack of theoretical and operative thoroughness in many dentists. The only implement needed in addition to the proper use of brush and floss is a small tube of nickeled brass bent at an angle of forty-five degrees at one end and intended to hold small shoe pegs. These points, straight and angle, can be used by the patient to wipe off the buccal and lingual cervix. The guide is the feel of the tooth and gum margin at the same time.

There is much difficulty in getting patients to do this, and they frequently backslide. Even the proper use of floss is hard to learn.

This negligence shows that patients like to do things in their own way and throw their responsibility for sins of omission upon the dentist, and they are apt to pin their faith to the brush and favorite antiseptics. This is, however, merely further proof that the obvious is the cause of their and our troubles, and that a reference to susceptibility is often an excuse with which we salve our consciences.

It may occur to us to ask why do teeth that are uncared for not decay while those that are brushed do decay?

We may answer the last question first, and say that teeth decay because they are not brushed thoroughly, as my former statements, which are capable of demonstration, show. We may answer the first by saying that teeth may not decay because they are not brushed. Miller demonstrated, in connection with his experiments in the production of artificial decay by the use of saliva and bread, that if the medium was not changed daily, putrefaction, with its alkaline reaction, would set in and decay would cease. If now, we think a moment, we can see that if an accumulation of bacteria and food occurs at some or many points about the teeth, a condition exactly analogous to the bread and saliva of Miller's experiments exist. Acid fermentation occurs. The acid exerts its chemical effect upon the enamel, but the food not being changed, the carbohydrate elements of the food are exhausted and all albuminous elements undergo putrefaction and the acid effects cease. Things remaining thus indefinitely, nothing in the way of decay occurs, but often calculus forms or pyorrhoetic effects occur. This may also throw light upon the fact that mouths that are pyorrhoetic are often free from caries.

We are fond of looking to the occult systemic condition in cases of pyorrhoea. I have seen bad cases allowed to get worse and worse when the obvious was so obvious that the breath told the tale almost before the eye took in the deplorable condition.

Let me instance two cases.

The first is of a lady who had been taking electrical treatment for rheumatism from a well-known specialist. Finding that he was not making headway he suspected a local supuration and referred the patient to a dentist for examination. The latter reported no pyorrhoea present. After five months of further rather unsuccessful treatment the patient was sent to me, and quite a number of deep pockets were found, each containing highly putrefactive material. The patient was not treated, as she was about to return to her home in the West, but directions for competent service were given her.

The second case was that of an out-of-town patient, apparently healthy. His dentist was a competent operator and very desirous of doing all possible for the patient.

A general pyorrhoea had gradually loosened several teeth slightly. The dentist treated it for two years with no success. The patient was told that no doubt a systemic condition underlay the pyorrhoea, and was referred to me.

There was no difficulty in determining the cause in small scales of calculus well under the gum margin of nearly every tooth, but in no case more than a quarter inch above it. The pus, of course, was due to an obvious infection.

After securing the promise of the patient to submit to systemic prophylaxis after treatment a general and thorough scaling was done and the patient dismissed for six weeks, the usual advice as to care being given but no systemic treatment advised. The case was a complete cure from the start, and no further trouble appeared until two years later a pocket was discovered on the lingual root of an upper molar. Recession had previously occurred, and in spite of prophylaxis no doubt some infection had occurred at this point only. It was treated locally and cured, but with difficulty.

It may incidentally be remarked that this patient would not use the wood point, and that the conformation of the tooth at this point presented the concavity between the lingual and mesio-buccal root as a catch-all for debris and bacteria.

Referring once more to the microbic plaques I may state that it is unreasonable to suppose that all plaques contain the same kinds or kind of bacteria and that presumably the species responsible for the peculiar extension of destructive bacteria (in plaques) into the pericimental tissue which brings about a pyorrhoea alveolaris differs from that or those that cause dental caries. The recent developments in pyorrhoea show this. There is no difficulty in demonstrating these plaques in proper position for action in any case of pyorrhoea. Again I must claim that pyorrhoea alveolaris is not an acute disease, but one requiring a long time. It begins, we may say, when the deposit of the plaque occurs, but it may be a long time, even years before the effects are seen as a distinct gingivitis with pockets.

To what extent the specialist is justified in claiming the existence of pyorrhoea in cases of unclean teeth may be an open question. The tendency seems to be to begin with any stain upon the teeth, at least I have been hurriedly consulted by two of my patients, who to my mind required a simple prophylaxis and nothing more, and in whose mouths not a vestige of gingivitis could be found. The specialist into whose hands they had fallen had quoted large fees for treatment of a suppositious pyorrhoea.

While the honesty of such methods may be questioned and incidentally the veracity of cures based upon such cases, there can be no doubt that the rank and file are neglectful of obvious cases of gingivitis, which if not cases of pyorrhoea, are the immediate forerunners, and they seem to be equally oblivious of the causes, namely the microbic plaques which, to my present way of thinking, are the real organic basis of serumal calculus of the subgingival variety which is associated with pyorrhoea.

I am not unmindful of the cases caused by malocclusion, but I feel sure that far more malocclusion is caused by pyorrhoea because the swollen membrane pushes the tooth from its alveolus or twists it around.

Also I am aware that many loose teeth and always those much loosened must be splinted and often shortened as they receive too much overwork ever to tighten up without splinting. I do not wish to go into the treatment of pyorrhoea, but aim here merely to call attention to the local causes, as opposed to the systemic.

It will not do to point to an aggravated case, one in its later stages after years of neglect, and neglect even though periodically treated, and say this must have an underlying systemic cause.

Let me here cite a case which might be laid down as due to systemic conditions.

About fourteen years ago a Jewish gentleman began to come to me about once a year, with marked pyorrhoea on one or two teeth and others threatening. The particular tooth was so badly loosened and the surrounding gum so swollen that extraction was its only treatment. This went on year after year until ten teeth were lost. No argument would convince the patient that frequent prophylaxis would avail. Finally the patient was convinced that it was the only hope of saving any teeth at all. For eight years the treatment has been instituted with intervals of from two to four months. No considerable amount of calculus has formed. No gingivitis beyond the slightest has supervened. No teeth have been lost and three bridges have been placed, thus adding extra work to the remaining pier teeth. These remain in as good condition as the rest.

Recently the patient remained away for six months. I sent for him, telling him as I often have done, that he could not afford to remain away. I found a calculus upon the right lower first molar in just the situation that I ordinarily find inspissated food masses which show he does not brush at that point. A slight gingivitis had begun at that point. The removal of the calculus removed the gingivitis.

This patient has had more or less indigestion regularly and is of bilious type. Why then does this not cause pyorrhoea in the intervals of cleansing? Also why does the pyorrhoea always follow the local cause and just where the cause is active? And why does any gingivitis so caused always clear up upon removal of the cause and stay cleared until the causes again accumulate?

If calculus or plaques are present for long periods and the disease results, the only systemic conditions that can be held in any way responsible are such as permit an excess of mucin or lime salts in the saliva or introduce a lack of resistance in the tissues to infection or introduce the infection itself or through its remedies as in the case of syphilis. The lack of resistance may be compared to the condition existing in acne pustulosa, in which a lowered resistance due to one of many causes complicates an infection of the skin.

The local treatment suffices in some cases, but where the infection is multiple and deep the resistance is raised by appropriate treatment or the opsonic power is raised by vaccines. Even here physicians are claiming that the constant effect of toxins produces the lowered resistance by exhausting the opsonic power. Such a lack of resistance is not to be denied in pyorrhoea, but that it or any definite condition can cause pyorrhoea alveolaris without the aid of local irritants as overwork of teeth, calculus and bacteria, or possibly the amoeba buccalis yet remains to be proven. That rare form of disease known as pericemental abscess beginning high up in the pericementum may be excluded here as its etiology is as yet obscure.

Once more are we not overlooking the obvious, and incidentally creating a class of specialists whose only reason for existence is our own inability to cope with a very simple proposition?

Within a year or more I have read this paper before several local societies and demonstrated the use of the implements. As a test I have informed them that I would furnish the name of the manufacturer of the point carrier if requested by mail to do so, coupling the offer with proof of my own lack of pecuniary interest. In the case of two societies the entire audience, though seemingly interested in the details, has failed to show any desire to obtain the implements, and I feel forced to the conclusion that as individuals, they are not personally interested in the application of its possible benefits to their patients, even to the extent of a trial of the method, and have even obtained expression from some that prophylactic suggestion would be of no value in their practice. Under these circumstances caries and pyorrhoea will no doubt continue unless a chemical specific be found which shall save the trouble of prophylaxis. To those who may care to take the trouble the following suggestions are offered:

(1) The brushing of the teeth with appropriate motions is valuable as far as it goes.

(2) In the use of the floss silk the patient should first rub tooth powder into the dental interspaces and then pass the floss gently down to and under the gum margin of one tooth without forcing into it so as to produce mechanical injury. The floss should then be bent as nearly as possible half way around that particular tooth and then with a slight sawing motion be slowly wiped over the entire approximal surface and part of the buccal and lingual as the floss is drawn toward the occlusal and out between the teeth. Next the floss is passed into the same space and the adjoining tooth cleansed. The performance of the act once a week should be supplemented by the use of a thin rubber band nightly as it is soft and less injurious mechanically than the floss.

For facilitating the introduction of floss into bridge spaces which can not be entered occlusally, the twisted wire bodkin is valuable. It is made by first forming from an old Gates Glidden drill, a button-hook shaped tool, the temper being of course first drawn. Over this hook is thrown a six-inch loop of fine regulating wire. The wire ends are held in the left hand close to the hook. The hook is slowly revolved in the engine hand piece and as the wire twists it is drawn through the fingers. The loose ends are cut off. Its chief virtue lies in the curve which may be given it best in line with the flat side of the eye. In use it is passed through any space and its curve causes it to return to the front of the mouth to be easily grasped and drawn through. The doubled floss is then used for the cleansing of all surfaces especially for the joint at the abutments. It is also useful for cleansing the lingual surfaces of fixed bridge work if properly formed.

Neither the floss nor the brush will cleanse the middle of the buccal and lingual cervix in many cases and for this defect the wood point is advocated.

The manner in which this is used is important. In all cases the carrier should be held in the hand as though using a pen after the manner of the Spencerian system of penmanship. The third and little finger are used as a fulcrum and rest upon the chin or teeth. The wood point is placed upon the distal cervix of the third molar just touching both the tooth and the gum. By rocking the hand slightly the wood point is then carefully drawn along the cervix until it rests in the first interspace, all jumping across the space being avoided. The position of the fulcrum fingers is then changed slightly and the next tooth is cleansed. The rule is to have the patient ''keep'' on the tooth but ''feel'' the gum as the work is done. All rubbing back and forth is to be avoided as this may injure the gum. The angle of the carrier permits this work to be done in all situations and once a week is enough.

It is not to be supposed that this self-treatment will take the place of a prophylaxis each three or four months by the operator, a thing that should be insisted upon by every dentist, in view of the modern tendency to pyorrhoea and caries. Some one has said that ''all patients are on their way to pyorrhoea if they have not already got it,'' and for this many dentists are responsible, for as shown by one case instanced in this paper, if some dentists do not know an advanced pyorrhoea when they see it others may not recognize the steps in that direction.

There is a great difference also in mouths that are ''kept'' in order and those that are ''put'' in order from time to time, say at intervals of one or two years.

The former system constitutes an easy, profitable and pleasure giving practice, while the latter is difficult, expensive to all concerned and eventually destructive of teeth as well as more or less unsightly despite the great skill involved and often employed. It seems to the writer better that patients should rather be proud of the care shown their teeth by themselves and their dentists than of the beautiful restorations and fine bridges in their mouths, no matter what credit the latter reflects upon the skill of their dentist. If this fact is not grasped by the profession into whose hands the coming generation shall fall then the Mouth Hygiene Movement will prove an abject failure except as an advertisement of dentistry. —*The Garretsonian.*

